# Data on the effect of conductive hearing loss on auditory and visual cortex activity revealed by intrinsic signal imaging

**DOI:** 10.1016/j.dib.2017.08.016

**Published:** 2017-08-31

**Authors:** Manuel Teichert, Jürgen Bolz

**Affiliations:** University of Jena, Institute of General Zoology and Animal Physiology, Erbertstraße 1, 07743 Jena, Germany

**Keywords:** Simultaneous intrinsic imaging, Auditory cortex, Visual cortex, Conductive hearing loss

## Abstract

This data article provides additional data related to the research article entitled “Simultaneous intrinsic signal imaging of auditory and visual cortex reveals profound effects of acute hearing loss on visual processing” (Teichert and Bolz, 2017) [1]. The primary auditory and visual cortex (A1 and V1) of adult male C57BL/6J mice (P120-P240) were mapped simultaneously using intrinsic signal imaging (Kalatsky and Stryker, 2003) [2]. A1 and V1 activity evoked by combined auditory and visual stimulation were measured before and after conductive hearing loss (CHL) induced by bilateral malleus removal. We provide data showing that A1 responsiveness evoked by sounds of different sound pressure levels (SPL) decreased after CHL whereas visually evoked V1 activity increased after this intervention. In addition, we also provide imaging data on percentage of V1 activity increases after CHL compared to pre-CHL.

**Specifications Table**

**Table:** 

**Subject area**	Neuroscience
**More specific subject area**	Cross-modal interactions
**Type of data**	Figures
**How data was acquired**	Optical imaging of intrinsic signals [2]
**Data format**	Analyzed
**Experimental factors**	Auditory and visual cortex activity was measured in adult mice before and after conductive hearing loss
**Experimental features**	Stimulus evoked cortical activity was measured using intrinsic signal imaging
**Data source location**	07743 Jena, Germany
**Data accessibility**	Data are provided within this article

**Value of the data**

• Demonstration that intrinsic imaging enables mapping of distinct sensory cortices simultaneously and investigation of cross-modal interactions.
• Data show that sound driven A1 activity decreases after conductive hearing loss whereas visually driven V1 activity increases after this intervention.
• Presented data may be relevant for understanding how an acute loss of one sensory modality can affect sensory procession in the remaining senses.
• The data provided here might stimulate further investigations on cross-modal integrations in early sensory cortices.

## 1 Data

**Fig. 1:**
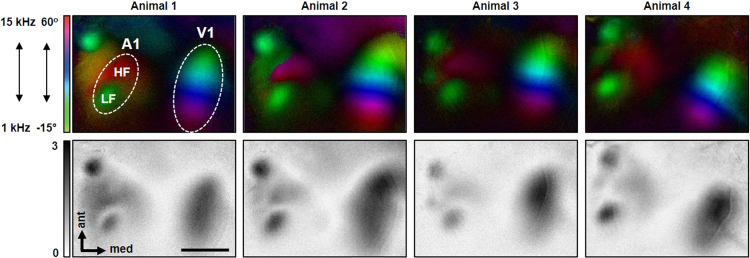
Simultaneous intrinsic imaging provides reliable maps of the auditory and visual cortex. The auditory stimulus was a tone sweep ascending or descending in frequency (1–15 kHz, 70 dB SPL) and the visual stimulus was a drifting horizontal light bar (100% contrast) presented in the binocular zone. Both stimuli were synchronized. Color and amplitude-coded polar maps are given (upper row) together with the corresponding grey-scaled amplitude maps. It is clearly visible that A1 appeared in two distinct regions: A green locus which is activated by deep frequency sound stimuli (about 1–5 kHz, LF) of 70 dB SPL and a red locos which is activated by high frequency sound stimuli (13–15 kHz, HF). The green locus in the V1 map represents the cortical area activated by binocular visual stimulation in the lower visual field whereas the red region represents the cortical area which is responsive to visual stimulation in the upper visual field. This tonotopic of A1 and visuotopic arrangement of V1 was found in all maps obtained by simultaneous intrinsic imaging. Scale bar: 1 mm.

**Fig. 2:**
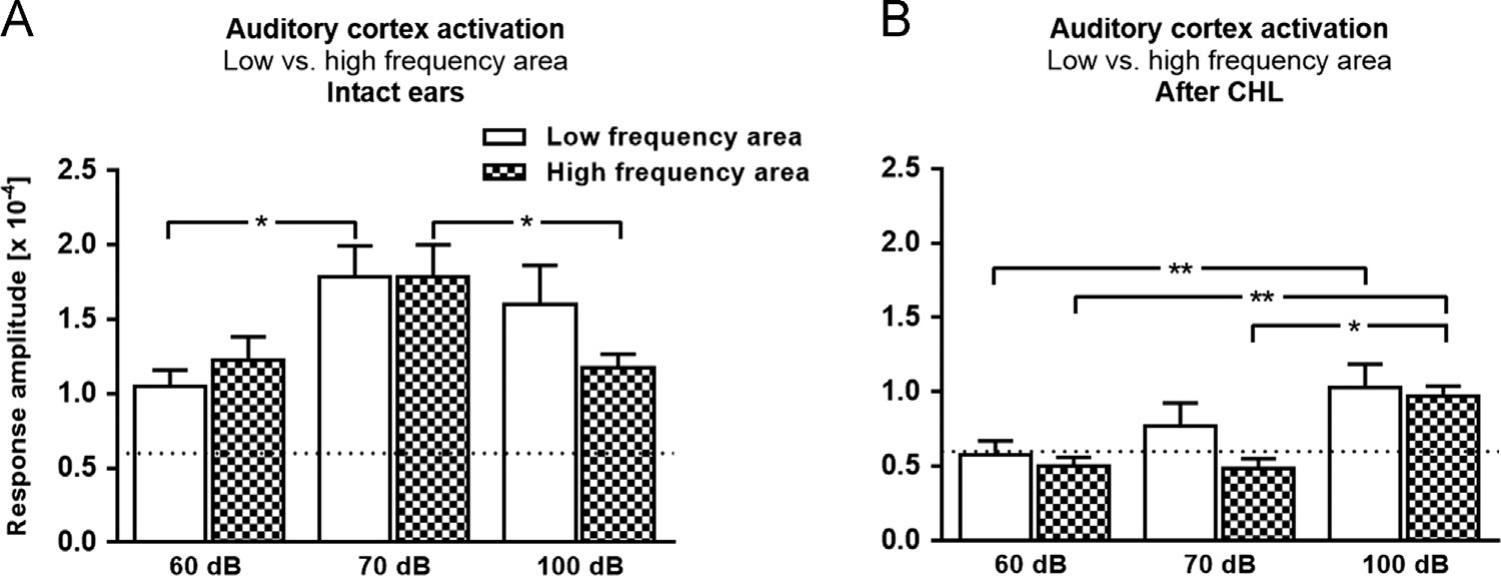
Data on the effects of auditory stimulation and CHL on sound driven activity of A1. (**A, B**) Using optical imaging of intrinsic signals we measured the responsiveness of the low and high frequency region of A1 before and after CHL induction. The auditory stimulus was a tone sweep linearly ascending or descending in frequency (1–15 kHz) with 60 dB, 70 dB or 100 dB SPL. Data are presented as means±s.e.m., *p<0.05, **p<0.01, ***p<0.001, paired and unpaired t-test. BG, background level (not responsive to auditory stimulation).

**Fig. 3:**
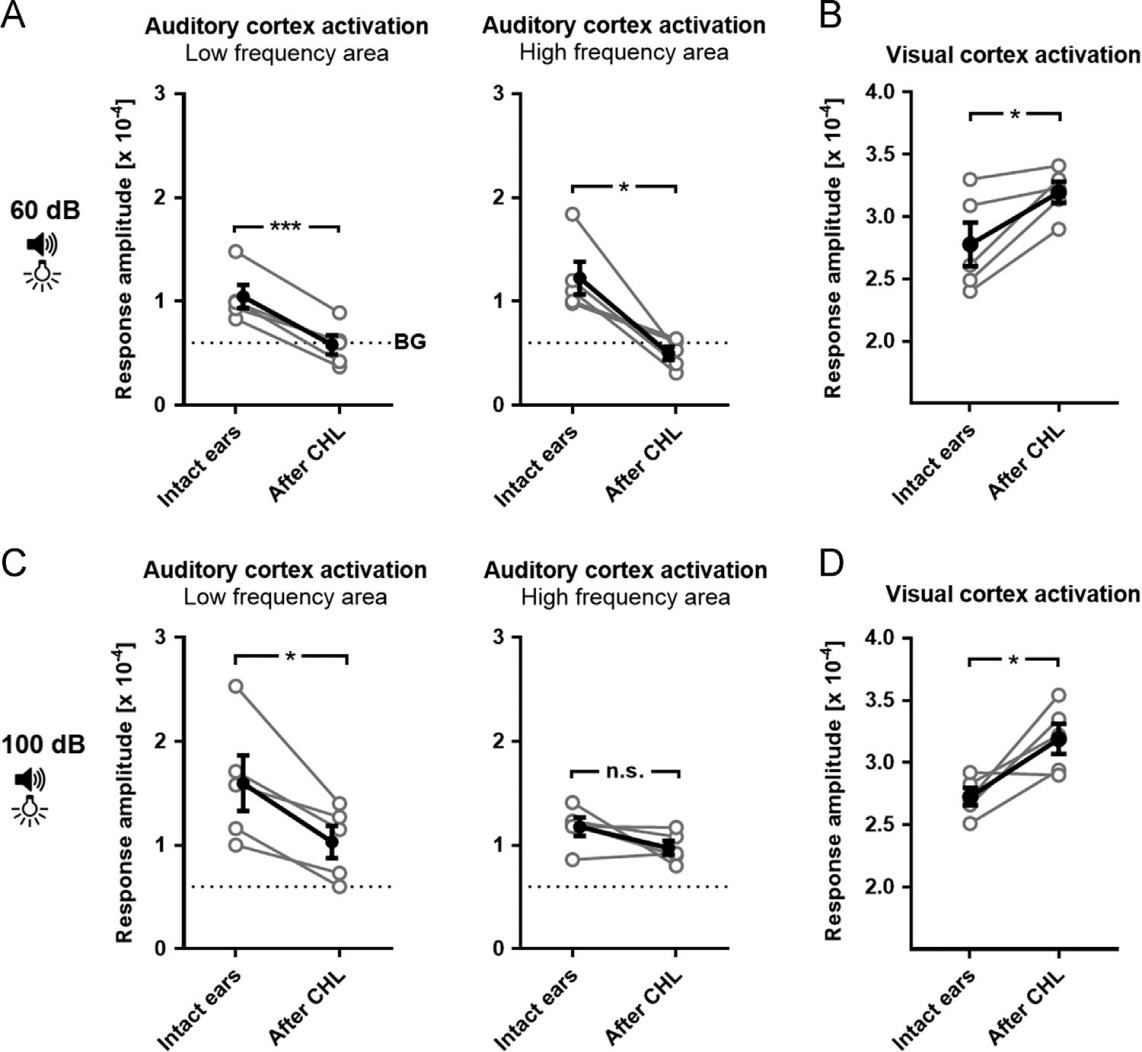
Dataset on the effects of CHL on sound driven A1 activity and visually driven V1 activity in individual animals. (**A, B**) We measured A1 and V1 responsiveness to combined auditory (60 dB SPL) and visual stimulation before and after CHL using simultaneous intrinsic imaging. The auditory stimulus was a tone sweep ascending or descending in frequency (1–15 kHz) and the visual stimulus was a drifting horizontal light bar (100% contrast) presented in the binocular zone. (**C, D**) A1 and V1 responsiveness before and after CHL. The stimulation was the same as described in A and B. However, the auditory stimulus had an SPL of 100 dB. Open circles show measurements of individual animals and closed circles show the mean of the open circles±s.e.m., *p<0.05, **p<0.01, ***p<0.001, paired t-test. BG, background level (not responsive to auditory stimulation).

**Fig. 4:**
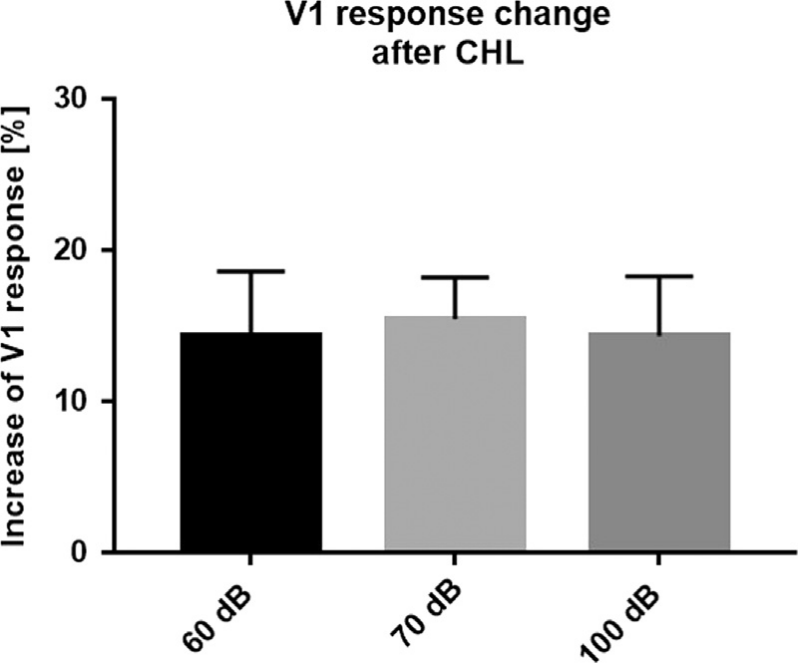
Data on the effects of CHL on V1 responsiveness. We measured V1 responsiveness to visual stimuli under concurrent auditory stimulation with 60 dB, 70 dB or 100 dB SPL before and after CHL. The auditory stimulus was a tone sweep ascending or descending in frequency (1–15 kHz) and the visual stimulus was a drifting horizontal light bar (100% contrast) presented in the binocular zone. We calculated the percentage increase of V1 responsiveness after CHL. Data are presented as means±s.e.m.

To investigate cross-modal interactions between A1 and V1, we developed a technique to simultaneously map A1 and V1 using periodic intrinsic imaging [1]. Using this novel approach we obtained reliable cortical maps of both sensory cortices (Fig. 1). As it has been shown that A1 activity effects V1 responsiveness [3], [4], we investigated the effects of an acute CHL on both A1 and V1 responsiveness. Therefore, we measured A1 and V1 responsiveness to combined visual and auditory stimulation before and after CHL in the same mice. First, we provide data of A1 activity in the low and high frequency regions of A1 elicited by auditory stimulation before and after CHL (Fig. 2**A, B**). Next we measured sound evoked A1 and visually driven V1 activity in individual mice under combined auditory (60 dB, 100 dB SPL) and visual stimulation before and after CHL. Fig. 3**A–D** provides a dataset showing changes of A1 and V1 activity after CHL. As a last step, we calculated the percentage increase of V1 responsiveness after CHL. For this, we measured visual driven V1 activity under concurrent auditory stimulation at 60 dB, 70 dB and 100 dB SPL and compared it to the V1 responsiveness under the same bimodal stimulation after CHL (dataset is given in Fig. 4).

## 2 Experimental design, materials and methods

### 2.1 Mouse preparation for optical imaging

Animals were initially anesthetized with 4% isoflurane in a 1:1mixture of O_2_/N_2_O and placed on a heating blanket (37.5 °C) for maintaining constant body temperature. Subsequently, mice received an injection of chlorprothixene (40 µg/mouse i.m.) and carprofen (4 mg/kg, s.c.). The inhalation anesthesia was applied through a plastic mask and maintained at 0.5% isoflurane during the experiment. Then, the animal was fixed in a stereotaxic frame. Next, we removed the skin of the left hemisphere to expose visual and auditory cortices. The exposed area was covered with 2.5% agarose in saline and sealed with a glass coverslip.

### 2.2 Optical imaging of intrinsic signals

#### 2.2.1 Imaging of visual and auditory cortex

Responses of mouse visual cortex were recorded as originally described by Kalatsky and Stryker (2003). Briefly, the method uses a periodic stimulus that is presented to the animal for some time and cortical responses are extracted by Fourier analysis. In our case, the visual stimulus was a 2° wide drifting horizontal light bar at 100% contrast and a temporal frequency of 0.125 Hz. The stimulus was presented on a high refresh rate monitor (Hitachi Accuvue HM 4921-D) placed 25 cm in front of the animal. Visual stimulation was adjusted so that it only appeared in the binocular visual field of the recorded hemisphere (−5° to +15° azimuth, −17° to +60° elevation). The stimulus was presented to both eyes for 5 min. Thus, it was repeated for about 35 times during one presentation.

For auditory stimulation, the stimulus was a tone sweep linearly ascending or descending in frequency in the range of 1–15 kHz with 60 dB, 70 dB or 100 dB sound pressure level (SPL) and a temporal frequency of 0.125 Hz. It was delivered by free field speakers placed 20 cm next to both ears and also presented for 5 min in each run.

For simultaneous imaging in both sensory systems we synchronized auditory and visual stimuli. In detail, as the bar started moving from the bottom of the monitor (−15°), the tone started at the same time with its lowest frequency (1 kHz). During the following 8 s the bar moved to the top of the monitor meanwhile the tone ascended linearly in frequency to 15 kHz. The synchronization was also maintained after the stimulus reversal. Background activity of A1 was measured in the region of A1 without sound stimulation. Calculation of background activity was performed in the same way like A1 map analysis (see Data analysis).

Using a Dalsa 1M30 CCD camera (Dalsa, Waterloo, Canada) with a 135×50 tandem lens (Nikon, Inc., Melville, NY) we first recorded images of the surface vascular pattern via illumination with green light (550±2 nm) and, after focusing 600 µm below the pial surface, intrinsic signals were obtained via illumination with red light (610±2 nm). Frames were acquired at a rate of 30 Hz and temporally averaged to 7.5 Hz. The 1024×1024 pixel images were spatially averaged to a 512×512 resolution.

#### 2.2.2 CHL induction between two imaging sessions

To measure the stimulus evoked A1 and V1 responsiveness before and after CHL in the same animals, we first performed one imaging session with intact ears followed by a subsequent session 5–10 min after CHL. After the first imaging session the anesthetized animal was removed from the stereotaxic frame. The eyes of the animal were protected with silicon oil. A CHL was always induced by bilateral malleus removal as described previously [5]. Briefly, the tympanic membrane was punctured and the malleus was removed under visual control through this opening using fine sterilized forceps [5], [6]. Great care was taken to avoid any destruction of the stapes and the oval window which is visible through the hearing canal (see [6]). After this surgery, the animal was re-fixed in the stereotaxic frame and the CCD imaging camera was re-adjusted and re-focused at the same position (like in the first session) above the region of interest, guided by the vascular pattern. The procedure usually took about 15 min. Subsequently, the next imaging session was started.

#### 2.2.3 Data analysis

From the recorded frames the signal was extracted by Fourier analysis at the stimulation frequency and converted into amplitude and phase maps using custom software [2]. For data analysis we used MATLAB [7]. In detail, from a pair of the upward and downward (ascending or descending, respectively) maps, a map with absolute visuotopy and tonotopy an average magnitude map was computed. The magnitude component represents the activation intensity of the visual or auditory cortex with darker pixels representing stronger activation. All magnitudes were multiplied with 10^4^ so that they can be presented in small numbers. To each condition (before or after CHL) we took at least three magnitudes of V1 or A1 responsiveness and averaged them for data presentation.

## 3 Statistical analysis

Optical imaging data were either compared by an unpaired *t*-test or paired *t*-test. The level of significance was set as *: *p*<0.05; **: *p*<0.01; ***: *p*<0.001. Data are presented as means and standard error of the mean (s.e.m.).

## Funding

This research did not receive any specific grant from funding agencies in the public, commercial, or not-for-profit sectors.
