# Supplementary material for: Data on the effect of conductive hearing loss on auditory and visual cortex activity revealed by intrinsic signal imaging
Source: Data Brief. 2017 Aug 31;14:659–64. doi: 10.1016/j.dib.2017.08.016 (PMC5591386; doi:10.1016/j.dib.2017.08.016)
Supplement: Supplementary file 1 — Supplementary material [file mmc1.pdf]

Conflict of Interest Statement

The authors declare that the research was conducted in the absence of any commercial or financial relationships that could be construed as a potential conflict of interest.

Sincerely,

Prof. Dr. Jürgen Bolz

Manuel Teichert
